# Investigational Paradigms in Downscoring and Upscoring DCIS: Surgical Management Review

**DOI:** 10.1155/2012/560493

**Published:** 2012-05-16

**Authors:** P. Orsaria, A. V. Granai, D. Venditti, G. Petrella, O. Buonomo

**Affiliations:** Division of Surgical Oncology, Department of Surgery, Tor Vergata University Hospital, 00133 Rome, Italy

## Abstract

Counseling patients with DCIS in a rational manner can be extremely difficult when the range of treatment criteria results in diverse and confusing clinical recommendations. Surgeons need tools that quantify measurable prognostic factors to be used in conjunction with clinical experience for the complex decision-making process. Combination of statistically significant tumor recurrence predictors and lesion parameters obtained after initial excision suggests that patients with DCIS can be stratified into specific subsets allowing a scientifically based discussion. The goal is to choose the treatment regimen that will significantly benefit each patient group without subjecting the patients to unnecessary risks. Exploring the effectiveness of complete excision may offer a starting place in a new way of reasoning and conceiving surgical modalities in terms of “downscoring” or “upscoring” patient risk, perhaps changing clinical approach. Reexcison may lower the specific subsets' score and improve local recurrence-free survival also by revealing a larger tumor size, a higher nuclear grade, or an involved margin and so suggesting the best management. It seems, that the key could be identifying significant relapse predictive factors, according to validated risk investigation models, whose value is modifiable by the surgical approach which avails of different diagnostic and therapeutic potentials to be optimal. Certainly DCIS clinical question cannot have a single curative mode due to heterogeneity of pathological lesions and histologic classification.

## 1. Introduction

### 1.1. Biopathological Profile

Ductal carcinoma in situ (DCIS) represents a heterogeneous group of proliferations varying in cytological and architectural appearance, for some of which it is believed that there are major clinical consequences [1]. Some studies have documented the sharing of molecular and genotypic characteristics in different benign and malignant stages of progression. Comparative analyses for implementing predictive markers in tumour biology show a multitude of genetic alterations in all the DCIS cases and propose distinct pathways in morphological evolution (poor, intermediate, and well). Poorly differentiated diseases displayed a higher frequency of amplifications (17q12, 11q13) and a higher average rate of genetic imbalances (40%) suggesting a developmental progression model for intraductal carcinoma [2]. Although the biological impact of these findings is not yet known, it is likely that DCIS differs by genetic grading and thus in prognostic implications. In fact the main question relating to the treatment is whether to consider DCIS a direct precursor of invasive cancer [3]. The natural history of small, noncomedo, and low grade in situ lesions treated by biopsy alone has been evaluated in studies with long-term follow-up. In the series reported by Sanders et al. 11 of 28 women (39.3%) have developed invasive breast carcinoma (IBC) after a median of thirty-one years, and 5 of 11 women (45%) died of metastatic disease [4]. In similar findings the risk of invasive disease has been described in a range of 14–75% of cases confirming a total progression impact of 43%. This has led to a rapidly consensus that atypical intraductal hyperplasia (AIDH) indicates a small, generalized, increased risk of breast carcinoma in both breasts that is approximately one half of low-grade DCIS lesions [5]. In our opinion, a practical difference between these diagnoses leads to a deeper level of understanding the rational therapeutics but it is also one of the most critical intersections of histopathology and clinical management today. With the diagnostic increase, the aim is to accurately identify clinically relevant lesions and streamline the treatment strategies. This emphasizes the significance of conceiving a less aggressive therapy (local excision only) when these lesions are limited in size, and the recurrence interval may well be beyond a reasonable life expectancy for the patient.

## 2. Evolving Knowledge

### 2.1. Critical Insights into Epidemiology

Percentage of carcinoma in situ (including DCIS and LCIS) from cancers diagnosed in the arm of selected screening studies now accounts for about 20–25% of all cases and from 17 to 34% of mammography detection [6].

In the United States, DCIS incidence rose from 1.87 per 100000 in 1973–1975 to 32.5 in 2004 [3]. Data from the Surveillance and End Results program depict about a 500% increase in DCIS among women aged 50 years and older from 1983 to 2003 with incidence starting to decline in 2003. An opposite trend has been verified among younger women in whom there has been a 290% increase since 1983 and the incidence continues to rise [7]. In addition, the prevalence of comedo subtype has not increased as rapidly as the less aggressive forms across all age groups. An analysis based on cancer registries found that between 1991 and 2001 the age-adjusted incidence of comedo DCIS was unchanged at approximately seven per 100000 rose from 16.5 to 31 per 100000 for noncomedo lesions [8]. The actual prevalence in the population is difficult to estimate because most patients are asymptomatic [7], but an improved understanding of information regarding frequency and risk factors could lead to critical insights into biological implications.

### 2.2. Classification Debate

Different classification systems may have important implications in clinical and prognostic management. No single scheme has been universally accepted and experts disagree as to which is the most appropriate [9].

The problem areas consist of the difficulties in separating low-grade DCIS from atypical intraductal hyperplasia (AIDH) and accurately defining disease size and extent. Moreover, due to the subjective interpretation of architecture and predictive features such as nuclear grade, necrosis, and polarization, many experienced pathologist differ in their diagnosis of DCIS [10].

According to the European Pathologist Working Group (EPWG) classification (G1, G2, G3), adopted by the European Organization for the Research and Treatment of Cancer (EORT), all the lesions are divided into three classes reflecting a statistically different association between nuclear grade and recurrence (*P*.009) [11]. 

The Van Nuys Prognostic index (VNPI) was developed to aid in the complex treatment selection process also including age, tumor size, and margin width to place patients in categories corresponding to clinical algorithms (Table 1). Pathological classification combines high nuclear grade and comedo-type necrosis to predict clinical behavior and stratify disease into three groups: non-high-nuclear grade without comedo-type necrosis (Score  1), or with necrosis (Score  2) and high nuclear grade (Score  3).

Silverstein et al. recently reported 31 local recurrences in 238 patients after breast-conservation surgery, 3.8% (3/80) in group 1, 11.1% (10/90) in group 2, and 26.5% (18/68) in group 3. The 8-year actuarial disease-free survivals were 93%, 84%, and 61%, respectively (all *P* ≤ 0.05) [1]. However, according to our experience, tissue processing by the Van Nuys protocol is complex, thus limiting its feasibility in clinical practice. 

Traditionally, highly heterogeneous intraductal proliferative lesions have been subdivided into noncancer—intraductal hyperplasia (IDH) and atypical intraductal hyperplasia (AIDH)—and cancer—DCIS, grades 1, 2, and 3. According to Tavassoli FA viewpoint a recognized problem with current classification is the interobserver variability and lack of reproducibility in lesions assignment with similar morphologic, immunohistochemical, and molecular characteristics. There is, for example, no justification in separating AIDH from low-grade DCIS because of their simply differences in size and quantity [10]. 

Moreover, the possibility of misunderstanding florid ductal hyperplasia is also really concrete. A review of 350 DCIS by expert breast pathologists resulted in a change in treatment recommendations in 93 (43%) cases and a conclusion that an expert assessment is necessary in this context [12].

According to Tavassoli the designation of carcinoma in situ is to be abandoned and it is necessary to unify the intraductal proliferations or alterations under the designation of ductal intraepithelial neoplasia (DIN) lesions that constitute risk factors for subsequent development of invasive carcinoma. There are three DIN categories, DIN-1 to DIN3. DIN-1 includes IDH (grade 1a), AIDH (grade 1b), and grade 1 DCIS (grade 1c); DIN-2 includes grade 2 DCIS; DIN-3 includes grade 3 DCIS [13]. 

In fact O'Connell et al. in the most comprehensive assessment of loss of heterozygosity (LOH) among intraductal proliferations showed that LOH of at least one genetic locus was shared with the synchronous invasive cancer in 37% of 19 patients with usual ductal hyperplasia (IDH), 45% of 11 patients with AIDH, 77% of 13 patients with non-comedo DCIS, and 80% of 11 patients with comedo DCIS [14].

In conclusion, DIN system offered a translational table for conversion of the currently used terminology of DCIS. This could lead to decrease impact of having two drastically different designations of cancer (DCIS) and noncancer (AIDH) applied to same lesions, caused by interobserver variability, reducing also geographically the term cancer-related overtreatment possibilities.

However, there are limitations to this classification, such as the inclusion of IDH among neoplasias, that may cause undue concern for those not aware that a tumor can be totally benign.

We think that analyzing with emphasis the areas of controversy and exciting new research prospects could enhance consistency in the interpretation and reporting of such complex and challenging disease.

### 2.3. Pathological and Predictive Features

In view of the increasing number of patients treated with breast conserving treatment (BTC) for ductal carcinoma in situ, risk factors for recurrence and metastasis should be identified (Table 2). 

The size of DCIS lesions has been correlated with LR but several studies have been criticized for performance in this regard.

Results by Ottesen et al. after 10-year follow-up reported a local recurrence (LR) rate of 11% and 48% for lesions smaller and larger than 10 mm, respectively, showing a significant association with a specific threshold [15]. However these findings were not supported by the French Cancer Centre's experience which identified LR rates of 30% and 31% in BCS group for lesions under or over 10 mm respectively, and 11% and 13% for the same subgroups in the BCS + RT group [16].

Surgical clearance is considered the most important risk factor for local recurrence and consensus has yet to be reached about optimal margin width. On univariate analysis Neuschatz et al. found that margin width and lesions size of initial excision specimens are significantly predicted for the presence of residual DCIS on reexcision. Residual tumor was found on reexcision in 41% of greater than 0-1 mm, 31% of greater than 1-2 mm, and 0% of greater than 2 mm clearance (*P* < 0001) [17].

In fact inadequate margins may result in high local recurrence, and excessively large resections may lead to poor cosmetic outcome without oncologic benefit.

In a recent meta-analysis when a 5 mm or greater margin was compared with a margin of 2 mm, no significance difference in the risk of IBTR (3.8% versus 5.8%) was observed (OR = 1.51; 95% CI, 0.51 to 5.04; *P* > .05). However, when specific margin threshold was examined, a 2 mm margin was found to be superior to a margin less than 2 mm (10.4%; OR 0.53; 95%CI, 0.26 to 0.96; *P* < .05) [18].

Women with high nuclear grade DCIS or clinical exhibition treated by lumpectomy may be appropriate candidates for additional treatment.

Studies of DCIS treated by BCS alone have reported LR rates ranging from 6% for low grade up to 31.5% for high-grade lesions [19].

Ottesen et al. supported the consideration of large cell/high grade DCIS as a biologically aggressive lesion with high recurrence rate and confirmed a low malignant potential with low failure rate at short-term follow-up and a delayed pattern of development for small and low type [15].

Histopathologically, in DCIS a strong association was found between large nuclear size and comedonecrosis as independent significant predictors. The recurrence rate among the high-grade/comedo-type lesions was 40%, 47%, 19%, and 33%, respectively, in different series treated by BCS alone [20–23]. The significance of comedo-type as a risk factor for LR has resulted in its inclusion in prognostic indices. The Van Nuys classification combines both features to define three distinct groups with predictive utility after BCS and facilitating clinical decision making [24].

## 3. Management

### 3.1. Clinical Practice

Based on the results of the several studies it is clear that DCIS represents a broad spectrum of disease and a uniform approach to treatment is not appropriate. Some patients require no treatment other than excision alone, others benefit from complete excision plus radiation therapy, and some will require mastectomy [25].

The challenge is using available clinical and pathologic data to define therapy for specific subsets of risk and quantify the evolving knowledge of prognostic factors.

Management strategies need to consider the breast and axilla, the need for adjuvant RT, and the utility of systemic adjuvant therapy. The gold standard in surgical treatment includes oncological radicality, optimizing cosmetic results with a positive psychological outcome. Today, through the joint activities of a multidisciplinary team and scientific expertise a uniform operating pattern is searched, but despite these general principles, the optimal management of DCIS remains controversial [5, 26].

### 3.2. Radical Treatment

Despite the significant transition from symptomatic patients toward those with screen detected pathology, paradoxically in some cases DCIS is managed with the radical intent applied to invasive breast cancer (IBC) [27]. Douek and Baum determined the impact of England screening on the type of surgery undertaken and reported an increase of 373% in the number of operations performed for DCIS and of 422% for the mastectomy practice over a period of 11 years [28].

In the French survey experience mastectomy (MX), conservative surgery alone (CS) and CS with radiotherapy (CS + RT) were performed in 30.5%, 7.8%, and 61.7% of 1289 patients, respectively (Table 3) [29].

Although the data indicate a sharp decline in the procedure rate, given the dramatic increase in the number of diagnoses, the actual incidence of MX at 7.8 per 100000 women did not change, and several studies confirmed an approximately application in one-third of cases. General guidelines recommend that patients with extensive or multifocal DCIS involving 4-5 cm of disease or more than one quadrant should be offered mastectomy. Moreover, women with potential contraindications to breast irradiation or a strong preference for mastectomy over breast conservation have been considered appropriate candidates for this procedure. The risk of a radical intervention is defined higher in some clinical scenarios like diffuse and suspicious-appearing microcalcifications, suboptimal tumor to breast size ratio with an unacceptable cosmetic results, the inability to obtain margin control by lumpectomy or reexcision(s) [30].

In the French survey the authors did not assess multifocality and multicentricity but analyzed the notion of residual tumor on the specimen in case of multiple surgery, maximal tumor size, and final margin status to predict the best surgical option (especially mastectomy). This study reported MX rates of 10% for lesions <10 mm compared to 72% for >20 mm, 11% for low-grade compared to 54% for high-grade lesions, and 43% for comedo carcinoma against 28% of other pathological subtypes [29].

The comparison of data in patients treated with MX and BCS showed a significant improvement in local control obtained with mastectomy (relapse free rate of 98.2% versus 89.7% at 10 years *P* = 0.02) without obvious impact on survival (98.7% in both groups) [31].

Thanks to the advances in diagnosis and improvements in reconstructive surgery, mastectomy will continue to be an important and acceptable treatment option in some cases. Cutulli et al. results confirm a 98% local control rate as reported by other series. After a 91-month median follow-up, local recurrence (LR) rates were 2.1, 30.1, and 13.8% in the MX, CS, and CS + RT groups of 716 women. The importance of case selection is discussed in relation to the high invasive recurrence rate following conservative surgery with (LR 59%) or without radiotherapy (LR 60%) and relative reported incidence of metastases reported in this subgroup (19%) [32] (Table 4).

Furthermore, among all surgically treated patients the cumulative risk of contralateral disease increased with an annual rate of 0.6% and some women undergo prophylactic mastectomy (CPM) to prevent cancer in the opposite breast. A recent surge reported a progression of CPM rate from 2.1% to 5.2% between 1998 and 2005, and factors contributing to this change most certainly include improved reconstructive outcomes and more widespread use of magnetic resonance [33].

Various approaches for radical surgery are currently used and include simple mastectomy (excision of breast tissue and overlying skin), skin-sparing approach (SMM), and, most recently, nipple-preserving techniques. In addition MX for DCIS is particularly suited to immediate breast reconstruction with an implant or autologous flap, as adjuvant RT and axillary involvement are less likely. The preservation of the natural skin envelope and inframammary fold during skin-sparing mastectomy would seem an ideal option to improve the aesthetic outcome of the instant reconstructive time, provided that clear margins are achieved. There has been a concern that it compromises the completeness of a mastectomy resulting in an increase in local breast cancer recurrence but large studies concluded that SSM or DCIS was an oncologically safe procedure with an LR rate similar to conventional MX (1–3%) [34].

The original Van Nuys prognostic index was created by combining lesion parameters and local recurrence as the markers of treatment failure. In the attempt to quantify the known important prognostic factors in DCIS, the USC/VNPI is offered as a guideline in a scientifically based discussion with the patient in order to define appropriate treatment. In Silverstein, patients with USC/VNPI scores of 10, 11, or 12 showed the greatest absolute benefit from postexcisional radiation therapy, but their LR rate continues to be extremely high and a recommendation for mastectomy should be considered [25]. In the future other factors like molecular markers may be integrated into the index to the extent that they are shown to be statistically important predictors of local relapse.

### 3.3. Breast Conserving Surgery

In spite of its often larger size, DCIS is a local disease lacking of two important components of the fully expressed malignant phenotype like stromal invasion and distant metastases. Its distribution is almost always segmental (unicentric) and complete excision is theoretically possible to achieve local clearance [24].

Faverly et al. have attributed the reliability of histological margin assessment to proliferation type, showing that continuous and multifocal growth pattern are usual in poorly and well differentiated in situ, respectively. However, in this series, only 8% of DCIS in 60 mastectomy specimens have a multifocal distribution with gaps greater than 10 mm, and this theoretically low likelihood of false free margin should encourage the use of conserving treatment for eradicable tumors [35].

Available data suggest that local control is optimized by the lumpectomy adequacy, regardless of the number of re-excisions required to achieve margin-negative status [36, 37]. Several investigators have also demonstrated that a diagnostic needle biopsy is associated with a higher success rate for subsequent BCS, improving single lumpectomy procedure results. The surgeon will plan a therapeutic partial breast resection with a more aggressive approach when the aim is to achieve margin control compared with when the goal is to sample adequately for a tissue diagnosis [38]. However, controversy remains regarding the oncological adequacy of BCS alone and the variable local relapse risk in randomized clinical trials evaluating DCIS treatment. In the National Surgical Adjuvant Breast Project (NASBP B-17) the overall recurrence rate for patients treated with excision only was 32% at 12 years, and 16% for patients treated with excision plus irradiation. At 4 years of follow-up, 9% of patients treated with excision plus radiation therapy had a local recurrence compared with 16% of DCIS treated with excision only in the EORT results, showing a statistically significant (approximately 50%) reduction in LR for patients who received RT [30].

Optimal local control is essential because in most reported series, approximately half of all local recurrences are invasive in each treatment group. In fact, breast preservation, with or without RT, yields a better cosmetic results but is accompanied by an increase in the probability of local failure.

The clinical value of recurrent DCIS is different from primary lesions and the prognostic implications of invasive disease are significant.

In particular the overall risk of metastasis has been reported to 0–3.6% for in situ LR, compared to 13.2–18% after invasive LR, and the axillary lymph node involvement with invasive LR is estimated from 11 to 30% [39, 40]. In Silverstein series the 8-year breast cancer-specific mortality and distant disease probability for 74 patients with LR previously treated for DCIS were 8.8% and 20.8%, respectively, while for the 35 invasive recurrences subgroup they were 14.4% and 27.1% [41].

Multivariate analysis showed that margin width, age, nuclear grade, and tumor size were all independent predictors of local recurrence (*P* < .001), with margin width as the single most important variable [42].

In 445 patients dataset with pure DCIS treated with excision alone, Heather at al. described the incremental benefit of larger margins. The median tumor size was 10 mm and after a median follow-up period of 57 months only 9 of 197 (4.6%) patients with a greater than 10 mm margin experienced local failure (Table 5). The probability of remaining free of local recurrences at 5 years was 93% without postoperative radiotherapy. The relative risk of developing an LR stratified by surgical margins was plotted as a continuous variable with a clear trend on decreasing the hazard ratio for local failure [43].

According to this approach, the most likely cause of local recurrence after excision alone for DCIS is inadequate surgery resulting in residual disease. In a previously reported data from 181 intraductal breast carcinoma, 76% of patients with initially involved margins had residual DCIS at mastectomy or reexcision, as did 43% of patients with initially clear margin (>1 mm) [44].

Neuschatz et al. analyzed reexcision specimens of 253 patients treated with lumpectomy for DCIS identifying residual disease in 63% of patients with transected margins, compared to 41% with greater than 0 to 1 mm, and to 31% with greater than 1 to 2 mm margins (Table 6) [17].

Yet one of the most important questions in the complex decision-making progress regards which patients selected for breast preservation require postexcisional radiation therapy. In our opinion, exploring the prognostic implications of histopathological features in BCS should be an excellent predictor of outcome, and with further corroboration, margin, width alone could possibly be used to determine the need for adjuvant RT in different risk subgroups.

The survival curves from the Van Nuys series showed that, regardless of the presence of high nuclear grade, comedonecrosis, large tumor size, or young age, the risk of local relapse remains slight if wide margins of resection are achieved. Consistent with the NSABP B-17 and EORTC trial findings, the absolute reduction of LR by RT increased with time from 7% at 4 years to 11% at 10.5 years but successive studies recognized that postoperative RT may not significantly improve the local outcome in all types of DCIS [27].

Di Saverio et al. confirmed the variable benefits of adding RT for different subsets of patients and so questioning the suitability of a uniform treatment policy. There was no advantage in the low VNPI score subgroup while it should be noted that in the groups with the higher VNPI score the benefit from adjuvant RT in avoiding local recurrence could be greater. Disease-Free Survival (DFS) at 10 years was 94.7% in CS compared to 92.3% in CS + RT in low VNPI (4-5-6) score group, 78.5% and 86.8% in the intermediate VNPI (5-6-7), and 50% against 100%, respectively, in the high VNPI (10-11-12) [31].

In the evaluation of USC/VNPI, Melvin et al. focused on the impact of margin status score on local recurrence. With margins 1–9 mm (score 2), there was a significant trend toward a benefit from irradiation. With margins less than 1 mm (score 3), there was a highly significant decrease in the probability of LR if radiation therapy was added [24].

These data suggest that margin width should be valued as an excellent predictor of local recurrence probability, and consequently, of the likelihood of residual DCIS. Silverstein et al. reported an 8% local recurrence rate for all conservatively treated DCIS lesions with margins of 10 mm or greater (VNPI score 1) [45] and Lagios and Silverstein showed a 5% local recurrence rate for all conservatively treated patients with the same margin status and lumpectomy alone compared to 4.5% in those treated by lumpectomy and irradiation [46].

In the selection process of pure DCIS cases, Van Nuys Prognostic system can be applied in conjunction with clinical experience to study tumor morphology and detection rate of local recurrence as the primary end points. Radiation therapy is not without side effects changing the texture of the breast and making subsequent mammography more difficult to interpret. Furthermore, its use may preclude the chance to implement a conservative treatment should it be needed in the future [47].

Consequently, subsets of patients who are not likely to receive any significant benefit from radiation therapy can be identified.

DCIS cases with VNPI scores of 3 or 4, low-grade lesions, small noncomedo lesions with uninvolved margins or well-differentiated lesions can be considered for treatment with excision only. This can be an important therapeutic cornerstone since such patients may account for more than 30% of the total number [48].

Patients with intermediate scores (5, 6, or 7) received a statistically significant 17% LR-free survival benefit when treated with radiation therapy (*P* = 0.017) but treatment recommendations for the intermediate group are the most difficult. DCIS cases with scores of 8 or 9, although showing the greatest relative benefit from RT, experienced LR rate in excess of 60% at 8 years and should be considered for mastectomy, generally with immediate reconstruction or reexcision if technically possible [41].

This controversy over the treatment selection may lead to a new conceptual approach on the operational chance of surgically modifying VNPI score and thus influencing the choice of more or less invasive strategy.

Potentially, in some cases, a patient can choose a reexcision, in order to downscore her lesion where the safety predictive criteria cannot be guaranteed. Successful downscoring of a patient with a USC/VNPI or 10 or 11 could result in substantial reduction in the risk of local recurrence, perhaps changing a recommendation from mastectomy to radiation therapy. Similarly, patients with close or involved margins with USC/VNPI scores of 7 or 8 after initial excision could opt for reexcision and a successful downscoring by achieving widely clear margins. This could result in a final score sufficiently low to avoid breast irradiation.

Moreover a dynamic surgical technique may be the basis for consolidating the diagnostic paradigms and further decrease the downtreatment risk.

In some case, reexcision will upscore the tumor, increasing the USC/VNPI by revealing a larger tumor size, a higher nuclear grade, the presence of previously undetected comedo necrosis, or an involved margin, suggesting in this way that mastectomy is preferable to select [25].

We deem prudent that the choice is acquired together, having surgeon to assist the patient to achieve his more or less conservative goal, according to the subjective and variable security needs and by counseling in a rational manner.

Moreover, due to the molecular heterogeneity of DCIS as well as the increasing trend toward individualized cancer treatment [49, 50], a Nomogram for predicting the risk of local recurrence after breast-conserving surgery published by Rudloff et al. integrates ten clinicopathologic variables to provide the probability of LR at 5 and 10 years after BCS. The risk estimate is specific on the individual patient and the final regression model was chosen according to the clinical and statistical significance of categorical variables like age at the time of surgery, family history, initial presentation, radiation therapy, adjuvant endocrine therapy, nuclear grade, necrosis, margins, number of excisions, time period of surgery, and their interdependent relationships. This tool may assist in individual decision making regarding various surgical or treatment options and help avoid over- and undertreatment of noninvasive breast cancer showing it to have favorable predictive accuracy and good model calibration [51].

Furthermore, we think that a more invasive procedure can be oriented on the axillary side as selective sentinel lymph node biopsy (SLNB) whose diagnostic mode has less aesthetic responsibility but a great role on therapy selection process.

In retrospective analysis of diagnostic procedures the invasion underestimation in needle biopsies of DCIS and the improvement in the sentinel practice have led some authors to support the lymphatic mapping at the surgery time [52]. In Lee et al. study there was no association between age, nuclear grade, tumor size, or presence of a mass in determining the likelihood of invasion, and of 59 patients diagnosed with DCIS by stereotactic biopsy, 29% were subsequently found to have invasive disease after surgery [53].

The status of the axillary lymph nodes remains the most powerful prognostic indicator of invasive breast cancer, but the role of axillary staging with SLNB for DCIS is controversial [54]. Nonetheless, surgeons have been removing lymph nodes in patients with a primary diagnosis of DCIS for a variety of reasons and with variable results [55]. This is consistent with findings from the recent French survey which reported overall rates of 21.3% for SLNB and 10.4% for axillary dissection (AD) [29]. Positive sentinel nodes have been reported in 0% to 13% of DCIS patients but among published studies, the incidence of SLNB metastasis among patients with initial diagnosis is substantially higher than among those with a final diagnosis like after excision or mastectomy (9.8% versus 5.0%) [3]. The information from axillary dissection or SLNB was of little value in the treatment of patients in whom no invasive cancer was found but it remains an attractive option when considering DCIS. The absolute indication for SLNB remains histological confirmation of concurrent or recurrent invasive disease. Therefore, in patients diagnosed with DCIS on core biopsy examinations SLNB should be reserved for those at high risk of invasive disease, including patients with palpable lesions, DCIS larger than 40 mm, high nuclear grade, comedo morphology, necrosis or recurrent disease, or patients undergoing mastectomy where SLNB could not be postponed [55].

Moreover axillary node involvement is higher with DCISM (5.1%) than that with DCIS (1.4%) and inadequate sampling could result in misdiagnosis and consequent under-treatment of patients [5]. The incidence rate of microinvasion among all DCIS cases is approximately 14% [56], and this theoretical foundation can support the significance of stratification risk in the practice of nodal spread staging procedure, in order to change the prognosis.

## 4. Conclusion

Since our knowledge of ductal carcinoma in situ (DCIS) continues to evolve, treatment decisionmaking has become increasingly complex and controversial for both patients and physicians. It represents a broad biologic spectrum of disease with a wide range of molecular heterogeneity, treatment approaches, and clinical recommendations but the need of local control should guide our decisions regarding therapy and help to risk stratify patients.

It is often difficult to justify mastectomy during an era of increasing practice of conservation for the more aggressive lesion (invasive breast cancer), but not all patients are candidates. The other side is to understand and define more accurately the application range of conservative treatment and its dynamic potential. Target is to treat patients effectively and decrease the risk of local recurrence, selecting the approach that will significantly benefit each patients group and not subject them to unnecessary risk. Specifically, the sophisticated questions that patients and doctors ask today are which subgroups of DCIS will benefit from postexcisional radiation therapy and how much and which can be treated by excision alone. Certainly the gold standard in clinical practice must be designed with an integrated approach to diagnostic and therapeutic features and functional skills aggregation. Nomograms are graphical depictions of predictive models that provide overall probability of a specific outcome for an individual patient, and in consultation with a physician, these tools can be used by patients to make decisions regarding various treatment options. The objective is to overcome the conflict between extent of resection, need of adjuvant strategies, and final aesthetic result with good oncological radicality.

## Figures and Tables

**Table 1:** The USC/VNPI scoring system.

Van Nuys Prognostic Index			
Parameter	Score 1	Score 2	Score 3
Van Nuys Classification	Group 1	Group 2	Group 3
	Non high nuclear grade without necrosis	Nonhigh nuclear grade with necrosis	High nuclear gradewith or without necrosis
Margins	≥10 mm	1–9 mm	<1 mm
Size	<15 mm	16–40 mm	>40 mm
Age	>60	40–60	<40

Modified from Silverstein; Ductal Carcinoma in situ of the breast 2nd ed. 2002.

**Table 2:** 

Author and reference	Parameter	Results			
Ottesen et al. [15]	Size	10-year LR rates of DCIS treated by BCS alone (*n* = 275)			
	<10 mm	LR 11%			
	>10 mm	LR 48%			

Cutuli et al. [29]	Size	5-year LR rates of BCS versus BCS + RT groups (*n* = 1,289)			
	<10 mm	LR 30%		LR 11%	
	>10 mm	LR 31%		LR 13%	

Dunne et al. [18]	Margin	Optimum margin threshold for DCIS resection (*n* = 2,514)			
Number of patients	Negative Margin Width	Percentage of patients with IBTR (5-year follow-up)			
914	No cells on ink	9.4			
1,239	1 mm margin	10.4			
207	2 mm margin	5.8			
154	≥5 mm margin	3.9			

Kerlikowske et al. [57]	Nuclear Grade	Invasive LR rates of DCIS treated by BCS alone (*N* = 1491)			

MacDonald et al. [43]		
	Low-grade lesions	6%			
	High-grade lesions	31.5%			

Silverstein et al. [1]	VNPI Score	LR rates and DFS in three groups of DCIS patients (*N* = 238)			
(1) Non-high-grade DCIS without comedo-type necrosis				3.8%	93%
(2) Non-high-grade DCIS with comedo-type necrosis				11.1%	84%
(3) High-grade DCIS with or without comedo-type necrosis				26.5%	61%

**Table 3:** Treatment modalities according 1289 DCIS patients.

Breast surgery	CS 7.8%	CS/RT 61.7%	MX 30.5%
	(France)	Range (84–96%)	Range (20–37%)
	(United States)	Range (39–74%)	Range (26–45%)

Axillary surgery	SLNB 21.3%	AD 10.4%	

Hormonal therapy	HT 13.4%		
	(France) range (6–34%)		

Reference [29].

**Table 4:** 8 years results of conservative and radical treatments in 716 DCIS.

	MX (145)	CS (136)	CS + RT (435)
Type of surgery	20.25%	18.09%	60.75%
8-year local recurrence rate	2.1% (3)	30.1% (41)	13.8% (60)
Noninvasive local recurrence	0% (0)	41.46% (17)	40% (24)
Invasive local recurrence	100% (3)	58.53% (24)	60.0% (36)
Nodal recurrence	0	3.7%	1.8%
Metastases	1.4% (2)	4.4% (6)	1.4% (6)

(All distant metastases occurred after previous invasive LR)			

Metastases among cases of invasive LR in CS and CS + RT 19% (12/60)			

Reference [32].

**Table 5:** Breast conservative surgery (BCS) results without RT.

Authors	Patients	Margin width	Local recurrence	Follow-up
Fischer et al. (2001) [58]	818	1 mm	31%	10 years
BiJker et al. (2001) [59]	1010	3 mm	13%	10 years
Houghtons et al. (2003) [60]	1701	1 mm	22%	4 years
Warren et al. (2005) [61]	1103		15%	7 years
Sabin et al. (2011) [62]	670	3 mm	High grade 18% Low grade 10.5%	7 years
Heather et al. (2005) [43] (median tumor size 10 mm)	197	>10 mm	4.6%	5 years

**Table 6:** Reexcision specimens analysis in patients treated with lumpectomy for DCIS.

	Margin Width (mm)	Residual Disease
(i) Silverstein et al. [44]	≥1 mm	43%
	<1 mm	76%
(ii) Neuschatz et al. [17]	0 mm (transected)	63%
	0-1 mm	41%
	1-2 mm	31%
